# Nationwide Study on Stress Perception Among Surgical Residents

**DOI:** 10.1007/s00268-022-06521-0

**Published:** 2022-03-18

**Authors:** Laura C. Guglielmetti, Christian Gingert, Anna Holtz, Reinhard Westkämper, Jochen Lange, Michel Adamina

**Affiliations:** 1 Department of Surgery, Cantonal Hospital Winterthur, Winterthur, Switzerland; 2 Faculty of Health, Department of Human Medicine, University of Witten/Herdecke, Witten, Germany; 3 Joint Private Practice for Orthopaedics, Traumatology & Surgery, Anna Und Nico Holtz, Kölnerstrasse 64, 42897 Remscheid, Germany; 4 Medkongress, St. Gallen, Switzerland; 5 Department of Biomedical Engineering, Faculty of Medicine, University of Basel, Allschwil, Switzerland; 6 MGA Network, Herisau, Switzerland

## Abstract

**Objective:**

Declining number of applicants and high attrition of residents are a dire reality. Surgeons in training are confronted to various stressors which interfere with their performance and may promote burnout. This study measures stress levels of Swiss surgical residents.

**Methods:**

Swiss surgery residents taking the Surgical Basic Exam from 2016 to 2020 completed the Perceived Stress Scale 10 (PSS). The PSS measures how unpredictable, uncontrollable, and overloaded the respondents evaluate their work life. Scores up to 13 are normal, and scores around 20 are highly pathologic. High subscores of helplessness (PH) and lower subscores of self-efficacy (PSE) indicate distress.

**Results:**

A total of 1694 questionnaires were evaluated (return rate 95.7%). Resident median (*m*) age was 29 years, 43.5% were female, and 72.7% of the residents were in their first 2 years of training. Residents reported a high PSS (*m* = 15), a high PH (*m* = 9), and an ordinary PSE (*m* = 5). Females reported worse PSS (*p* < 0.001), PH (*p* < 0.001), and PSE (*p* = 0.036). In multivariable analysis, male sex (*p* < 0.001), aiming at orthopedic (*p* = 0.017) or visceral surgery (*p* = 0.004), and French as mother tongue (*p* = 0.037) predicted lower stress levels, while graduating from a country not adjacent to Switzerland led to higher stress (*p* = 0.047).

**Conclusion:**

Perceived stress levels are high in this prospective and representative cohort study of Swiss surgical residents. Females endured significantly worse stress and helplessness levels than males. These figures are worrisome as they may directly contribute to the declining attractivity of surgical residencies. Detailed sex-specific analysis and correction of stressors are urgently needed to improve residency programs.

## Introduction

Stress commonly describes the mental, emotional, or physical reaction caused by internal and external stimuli. Depending on the individual reaction, stress factors may cause positive (eustress) or negative (distress) stress [1]. If a situation is evaluated as overwhelming, uncontrollable and impossible to cope with, distress inevitably occurs [2]. The impact of chronic stress on all aspects of life has been studied extensively, including physical and psychological health, workplace performance, learning, and even mortality [3–6]. While there is a common understanding of the negative effects of chronic stress, its origin has various reasons, which are intensively researched. Consequently, many different assessment forms exist with emphasis on a biological, environmental, or psychological origin or a combination thereof [7–11].

The psychological approach focuses on individual appraisal of stress and reflects the individual significance of stress factors and the coping skills at hand [12]. The Perceived Stress Scale (PSS), a self-reporting measurement, was developed and published by Sheldon Cohen in 1983 [3]. It is one of the most frequently used and well-established psychological instruments for measuring perceived stress. The 10-item questionnaire (PSS-10) is designed to assess how unpredictable, uncontrollable, and overloaded the respondents value their work lives over the past month. The final result consists of two subscores, perceived helplessness and perceived self-efficacy (the ability to cope) [13]. The PSS-10 instrument has been translated in various languages, and it was used in a multitude of populations and cultures [14]. It is considered applicable for the majority of human populations and its different subgroups. In particular, both the German and French versions were extensively validated [15, 16]. Furthermore, it requires little time to be completed [17].

High levels of stress may occur within the healthcare environment. Countless studies have shown the negative impact of stress exposure on medicine, pharmacy, and nursing students and professionals [18, 19], including depression and suicide thoughts affecting medical students and physicians [20, 21]. However, very few studies have focused on surgical residents. Importantly, behavioral career patterns are often established in the first years of surgical training. Within this decisive time window, young surgeons are exposed to high expectations and various stressors, which may lead to gradual exhaustion, interfere with their clinical performance, and result in a burnout and residency attrition [22]. Hence, understanding and mitigating stress in surgical residents is critical to the maintenance of the surgical workforce with an expected shortage of tens of thousands of surgeons within a decade in Europe and North America [23].

Considering the steadily declining number of applicants for surgical specialties in Switzerland and Europe and a worrisome attrition rate in North America [24–28], investigations of the causes of the observed disenchantment, including the impact of the learning and workplace environments, are urgently needed [29–33].

The present study aims at measuring the perceived stress levels of surgical residents in Switzerland, considering sex, age, nationality, and country of graduation.

## Methods

### Participants and setting

Swiss residents who took the first step examination of surgical qualifications between 2016 and 2020 were offered to voluntarily and anonymously complete the PSS-10 either in its validated German or French translations while registering for the examination. Medical school is taught in German or French in Switzerland, while 90% of the hospitals operate in German or French and 10% in Italian.

Swiss board certification in a surgical discipline requires a two-part assessment. Passing the first part of the specialty examination is mandatory for residents in general surgery, thoracic surgery, cardiac surgery, vascular surgery, orthopedics, hand and plastic surgery, pediatric surgery, maxillofacial surgery, and urology. Residents are advised to take the written examination once they have completed their first 2 years of training (common trunk). The test focuses on medical and surgical knowledge common to all surgical disciplines, including perioperative management, elective and emergency procedures, and basic knowledge of oncology, immunology, infectiology, pathophysiology, law, and ethics. Once the Surgical Basic Exam has been passed and the 2-year common trunk completed, residents may opt for specialized training, e.g., urology or cardiac surgery, or stay in a broader discipline, e.g., general surgery or orthopedics. Residents then graduate after 4 more years of training and passing the second part of the specialty examination to become board certified.

### PSS-10

The PSS is a validated tool to measure how unpredictable, uncontrollable, and overloaded the respondents evaluate their work life over the last month [3, 13–16, 34–36]. Each of the ten items is rated on a five-point Likert scale from 0 (never) to 4 (very often). Hence, individual scores can range from 0 to 40. A high PSS score indicates a high level of stress, with scores around 13 considered as average and scores around 20 as highly pathologic [36, 37]. Two subscores are included in the PSS [38, 39]: perceived helplessness (PH) and perceived self-efficacy (PSE, which has a reverse scale: High value indicates low level of perceived self-efficacy). PH emphasizes the individual’s reaction to stress, while PSE denotes the self-assessed ability to cope with these stressors. A high PSS score as well as a high “helplessness” subscore indicates distress, while a high “self-efficacy” subscore represents a lower ability to cope with stressors [15].

### Statistical analyses

Descriptive statistics were used to summarize patients’ characteristics. Normality was assessed using graphical methods (Q–Q plots and histograms) and the Shapiro–Wilk test. Continuous variables were reported as mean and standard deviation (SD) or median and interquartile range (IQR) and compared between the two groups using two-sample independent t-tests or Mann–Whitney U test according to data distribution. For comparisons between more than two groups, the Kruskal–Wallis test was applied with post hoc analyses (Dunn’s post hoc test) of the subgroups and subsequent Bonferroni correction for multiple comparisons.

Paired continuous variables were compared using paired-samples *t*-test.

Categorical variables were summarized as frequencies (%) and compared using Pearson’s Chi-squared test or Fisher’s exact test where applicable. In cases of ≥10% missing information for a variable, the number of participants with complete information is reported.

Age was categorized as 5-year intervals (<25, 26–30, 31–35, 36–40, >40).

Multivariable linear regression analyses were conducted with PSS-10 score, PH score, and PSE score as dependent variables and age, sex, specialty aspired, position, native language, and duration of training as independent variables. Variable selection for univariable analysis was a priori determined based on clinical parameters and literature review [36, 40, 41]. Variables with a p-value less than 0.10 in univariable analysis were retained in the multivariable models.

Categorical variables were coded as dummy variables. Independent variables were entered simultaneously.

SPSS version 25 (IBM Corp., Armonk, NY) was used for data analysis. *P*-values ≤ 0.05 are considered statistically significant.

### Ethical approval

Examinees taking the first part of the board examination and queried in the present study agreed to the use of their personal data. This study was approved by the regional ethical board (Ethikkommission Nordwest- and Zentralschweiz, Swissethics study ID 2019-00167). Data management was compliant with the Swiss Federal Data Protection Act.

## Results

Out of 1770 distributed questionnaires over the study period of 5 years, 1694 (95.7%) were returned and included in the current analysis. Men accounted for 56.5% and women for 43.5% of the responders with a median age of 29 years (IQR 27.6–31.0 years). Distribution of the demographic characteristics of the responders did not differ from the overall examination takers. Median duration of training of all participants was 12 months (IQR 4–24), whereas 27% (*n* = 460) of the participants had more than two years of training. Orthopedic surgery (*n* = 406, 24%) and general surgery (*n* = 402, 23.8%) were the two specialties most sought for.

Men were slightly older (median 29.2 years, IQR 27.8–31.2 years) than women (28.7 years, IQR 27.2–30.1 years), *p* < 0.001. Males aimed predominantly at a specialty in orthopedic surgery (*n* = 285, 40.5%), while females preferred general surgery (*n* = 209, 41.1%). Only 38.7% of the surgical residents in Switzerland graduated from a Swiss medical school. Baseline demographics of the overall cohort are presented in Table 1.

**Table 1:** Demographics of the cohort of Swiss surgical residents

*N* (%) unless stated otherwise	Total *n* = 1694	Male * n* = 942	Female *n* = 726	*p*-value
Median age (years), (IQR)	28.95 (27.6–31.0)	29.16 (27.77–31.23)	28.70 (27.23–30.71)	< 0.001
*Known for n = 1524 participants*				0.006
≤ 25 years	14 (0.8)	3 (0.3)	11 (1.7)	
25.01–30 years	959 (56.6)	530 (61.3)	428 (65.1)	
30.01–35 years	429 (25.3)	250 (28.9)	179 (27.2)	
35.01–40 years	99 (5.8)	66 (7.6)	33 (5.0)	
≥40 years	23 (1.4)	16 (1.8)	6 (0.9)	
*Year of examination*				0.14
2016	299 (17.7)	167 (17.7)	118 (16.3)	
2017	280 (16.5)	157 (16.7)	116 (16.0)	
2018	253 (14.9)	144 (15.3)	104 (14.3)	
2019	446 (26.3)	262 (27.8)	184 (25.3)	
2020	416 (24.6)	212 (22.5)	204 (28.1)	
Aim				
*Known for n = 1234 participants*				0.226
Surgery	402 (23.8)	184 (26.2)	209 (41.1)	
Orthopedics	406 (24.0)	285 (40.5)	112 (22.0)	
Urology	102 (6.0)	74 (10.5)	27 (5.3)	
Plastic Surgery	95 (5.6)	46 (6.5)	48 (9.4)	
Visceral Surgery	57 (3.4)	30 (4.3)	27 (5.3)	
Hand Surgery	33 (1.9)	17 (2.4)	16 (3.1)	
Pediatric Surgery	31 (1.8)	16 (2.3)	15 (3.0)	
All others	107 (6.3)	51 (7.3)	54 (10.6)	
Position				0.798
*Known for n = 1681 participants*				
Resident	1612 (95.2)	896 (95.2)	691 (95.2)	
Chief resident	31 (1.8)	16 (1.7)	15 (2.1)	
Research	38 (2.2)	23 (2.4)	14 (1.9)	
Medical school				0.433
*Known for n = 838 participants*		N = 467 (100%)	N = 370 (100%)	
Switzerland	324 (38.7)	183 (39.2)	141 (38.1)	
Germany	182 (21.7)	85 (18.2)	97 (26.2)	
Austria	163 (19.4)	99 (21.2)	63 (17.0)	
Italy	85 (10.1)	53 (11.3)	32 (8.7)	
France	84 (10.0)	47 (10.1)	37 (10.0)	
All others and unknown	791 (46.7)	453 (49.2)	337 (47.7)	
*Nationality*				< 0.001
Swiss	662 (39.1)	378 (40.7)	283 (39.9)	
German	315 (18.6)	146 (15.7)	168 (23.7)	
Austrian	83 (4.9)	53 (5.7)	30 (4.2)	
Italian	162 (9.6)	108 (11.6)	54 (7.6)	
French	77 (4.5)	45 (5.0)	31 (4.4)	
All others	342 (20.2)	197 (21.1)	143 (20.2)	
Mother tongue				
*Known for n = 1231 participants*				0.313
German	666 (39.3)	373 (51.9)	291 (57.2)	
French	212 (12.5)	129 (18.0)	82 (16.1)	
Italian	149 (8.8)	96 (13.4)	53 (10.4)	
Spanish	21 (1.2)	14 (1.9)	7 (1.4)	
All others and unknown	183 (10–8)	106 (14.8)	76 (14.9)	
Swiss national mother tongue versus other native language	1371 (83.4)	777 (83.5)	594 (83.3)	0.894
Median duration of training (months) (IQR)	12 (4–24)	12 (6–24)	12 (2–24)	0.019

*IQR* interquartile range, *Medschool* country of graduation from medschool

Median PSS-10 score of the overall cohort was 15.0 points (IQR 11.0–19.0) with subscores of PH of 9.0 (IQR 6.0–13.0) and of PSE of 5.0 points (IQR 4.0–7.0). In univariate analysis, female sex was uniformly associated with worse stress levels across all dimensions. Aiming at a specialty in general surgery, having graduated in Italy and/or being an Italian was also related to worse stress scores, whereas being a French/having French as a native language and aspiring to a specialty in visceral surgery was protective. Last, stress scores were not affected by the duration of surgical training or the hierarchical position of the resident. Table 2 summarizes the PSS-10, the PH, and the PSE scores of the overall cohort and for several subgroups of the study population.

**Table 2:** Perceived stress, perceived helplessness, and perceived self-efficacy according to demographic characteristics

Median, IQR	PSS 10 score	*p*-value	PH score	*p*-value	PSE score	*p*-value
Overall cohort, *n* = 1694	15 (11–19)		9 (6–13)		5 (4–7)	
Males, *n* = 941	14 (10–19)	<0.001	9 (6–12)	<0.001	5 (4–7)	0.036
Females, *n* = 726	15 (11–19)		10 (7–13)		5 (4–7)	
*Age groups*		0.5		0.028		0.675
≤25	16 (10.8–18)		9 (5.8–12.3)		6 (4.8–7)	
25.01–30	15 (11–19)		10 (7–13)		5 (4–7)	
30.01–35	14 (10–19)		9 (6–12)		5 (4–7)	
35.01–40	15 (9–19)		8 (5–12)		5 (3–8)	
≥40	13.6 (9.5–18)		8 (4–11.3)		6 (4–8)	
*Aim*		0.031		0.101		0.048
Surgery	16 (12–20)		10 (7–13)		6 (4–7)	
Orthopedics	14 (10–19)		9 (6–12)		5 (4–7)	
Urology	15 (10.3–19)		10 (6–13)		5 (4–6.5)	
Plastic Surgery	15 (11.5–19)		9 (6–13)		5.5 (4–7)	
Visceral Surgery	13 (9.5–20.8)		8.5 (6.8–13.3)		4.5 (3–7)	
Hand Surgery	17.5 (14–22)		11 (9.8–14.8)		6 (4–8)	
Pediatric Surgery	15 (10–19.5)		9 (5–13)		5 (4–7)	
All others	15.5 (10–20)		10 (6–13)		5 (4–8)	
*Position*		0.254		0.246		0.266
Resident	13 (7–17.8)		9 (2.3–12.8)		4 (4–5)	
Chief resident	12 (7–18)		7 (4–12)		5 (3–6.5)	
Research	15 (11–19)		9 (6–12.5)		6 (4–7)	
*Medical school*		0.205		0.428		<0.001*
Switzerland	14 (11–18)		9 (7–13)		5 (3–6)	
Germany	14 (10–19)		9 (6–13)		5 (3–7)	
Austria	15 (9–19)		10 (6–12)		5 (3–6)	
Italy	16 (10–20)		10 (5.3–13)		5.5 (4–7)	
France	14 (10.3–18.8)		8.5 (5–11)		5 (4–7)	
All others	15 (11–19)		10 (6–12.3)		5 (4–7)	
*Nationality*		0.01		0.002		<0.001****
Swiss	15 (11–19)	**	10 (6–13)	***	5 (4–7)	
German	15 (11–20)		10 (7–14)		5 (4–7)	
Austrian	13 (10–18)		9 (6–12)		5 (3–6.3)	
Italian	17 (13–20)		10 (7–13)		6 (5–8)	
French	13.5 (8.3–18)		8 (5–12)		5 (3–7)	
All others	14.5 (10–19)		9 (6–12)		5.5 (4–7)	
*Mother tongue*		0.011		0.002		<0.001*******
German	15 (11–19)	*****	10 (7–13)	******	5 (4–7)	
French	14 (10–18)		8 (5–11.5)		5 (4–7)	
Italian	16 (13–20)		10 (7–12)		6 (5–8)	
Spanish	14.5 (11.3–19.5)		8.5 (5.3–9.8)		6.5 (5–9.8)	
All others	16 (11–21)		10 (6–13)		6 (4–8)	
Swiss national mother tongue versus other native language		0.691		0.466		0.04
	15 (10–20)		9 (6–13)		6 (4–8)	
	15 (11–19)		9 (6–13)		5 (4–7)	
*Duration of training*		0.311		0.128		0.062
≤12 M	15 (10–19)		9 (6–13)		5 (4–7)	
12.01–24 M	15 (12.19.8)		10 (7–13)		5 (4–7)	
24.01–36 M	15 (11–19)		9 (7–13)		5 (4–8)	
36.01–48 M	15 (9.75–20)		9 (5–12)		5 (3.75–7)	
48.01–60 M	17 (8–20)		8 (6–13)		6 (4–8)	
60.1–72 M	13 (7.5–18.5)		6 (4–12.3)		5 (4–7.5)	
>72 M	13 (8–17)		8 (4–12)		5 (4–7.5)	

*p-value after Bonferroni correction for multiple comparisons: Austria versus all others p = 0.009, Germany versus all others p = 0.012, Switzerland versus all others p = 0.002

**p-value after Bonferroni correction for multiple comparisons: French versus Italian p = 0.030

***p-value after Bonferroni correction for multiple comparisons: French versus Italian p = 0.048, French versus German p = 0.009, all others versus German p = 0.040

****p-value after Bonferroni correction for multiple comparisons: Austrian versus Italian p = 0.001, Swiss versus Italian *p* < 0.001, German versus Italian *p* < 0.001

*****p-value after Bonferroni correction for multiple comparisons: French versus Italian *p* = 0.007

******p-value after Bonferroni correction for multiple comparisons: French versus German *p* = 0.001

*******p-value after Bonferroni correction for multiple comparisons: German versus all others *p* = 0.035, German versus Italian *p* < 0.001, French versus Italian *p* = 0.011

*IQR* interquartile range, *M* months, *Medschool* country of graduation from medschool

When compared to a contemporary, age-matched reference group of the general population [15], Swiss surgical resident demonstrated significantly worse summative PSS scores (15. 0 vs. 12.74, *p* < 0.001) and PH (9.0 vs. 7.6, *p* < 0.001). This observation holds true for both men and women. Noteworthy, Swiss residents scored better than the reference population in PSE (M 5.0 vs. M 5.8, *p* < 0.001).

In multivariable regression analysis, male sex independently predicted better PSS-10 (β = −1.201, *p* < 0.001) and PH score (β = −1.154, *p* < 0.001) when controlled for age (PH score only), specialty, nationality, country of graduation, hierarchical position, native language, and duration of training. Longer duration of training was an independent predictor for decreasing levels of PSS and PH, reaching statistical significance when the last year of residency was compared to the end of the common trunk (12–24 months of training).

Residents in orthopedic (β = −1.119, *p* = 0.003) and visceral surgery (β = −2.080, *p* = 0.001) and those who had more than 5 years of training (β = −2.190, *p* = 0.017) were less stressed out and were less prone to helplessness. Similarly, perceived self-efficacy was better in residents training in orthopedics, urology, and visceral surgery.

Conversely, Italian citizenship and native language, graduating from a country not adjacent to Switzerland, and one year or less of training exposed to higher stress and lower self-efficacy. Further, perceived helplessness was greater in German citizens and lower for the French national, French native speaker and graduate, and for those with more than 5 years training. Detailed results of all regression models are reported in Tables 3, 4, and 5.

**Table 3:** Uni- and multivariable linear regression analysis of perceived stress scores with sex, age, intended specialty, nationality, country of graduation, position, native language, and duration of training as independent variables

	B-coeff	95% CI		*p*-value	B-coeff	95% CI		*p*-value
		Lower	Upper			Lower	Upper	
Male sex	−1.251	−1.858	−0.645	<0.001	−1.201	−1.827	−0.575	<0.001
*Age*	−0.02	−0.112	0.069	0.645				
≤25 years	−0.315	−3.462	2.831	0.844				
25.1–30 years	Ref	Ref	Ref	Ref				
30.1–35 years	0.032	−1.216	1.28	0.96				
35.1– > 40 years	−0.499	−1.666	0.667	0.401				
*Aim*								
Surgery	Ref	Ref	Ref	Ref	Ref	Ref	Ref	Ref
Orthopedics	−1.119	−1.836	−0.376	0.003	−0.938	−1.709	−0.168	0.017
Urology	−1.163	−2.322	−0.003	0.049	−0.712	−1.886	0.463	0.235
Plastic Surgery	−0.651	−1.869	0.567	0.294	−0.762	−1.981	0.457	0.22
Visceral Surgery	−2.08	−3.35	−0.811	0.001	−1.843	−3.114	−0.573	0.004
Hand Surgery	0.895	−0.976	2.767	0.348	1.174	−0.705	3.053	0.221
Pediatric Surgery	−1.143	−2.957	0.672	0.217	−1.08	−2.891	0.731	0.242
Other	−0.378	−1.632	0.877	0.555	−0.274	−1.549	1.001	0.673
*Nationality*								
Swiss	Ref	Ref	Ref	Ref	Ref	Ref	Ref	Ref
German	0.688	−0.155	1.531	0.11	−0.192	−1.231	−1.231	0.718
Austrian	−0.774	−2.193	0.645	0.285	−1.413	−2.954	−2.954	0.072
Italian	1.335	0.238	2.431	0.017	−0.391	−2.185	−2.185	0.669
French	−1.154	−2.59	0.281	0.115	−0.872	−2.591	−2.591	0.32
Other/unknown	0.14	−0.646	0.927	0.726	−0.936	−2.102	−2.102	0.115
*Medical school*								
Switzerland	Ref	Ref	Ref	Ref	Ref	Ref	Ref	Ref
Germany	−0.238	−1.372	0.896	0.681	−0.336	−1.523	0.851	0.579
Austria	−0.296	−1.481	0.889	0.625	−0.232	−1.438	0.974	0.706
Italy	0.726	−0.787	2.239	0.347	1.383	−0.303	3.07	0.108
France	−0.545	−2.091	1.001	0.489	0.17	−1.57	1.911	0.848
Others/unknown	0.798	0.001	1.595	0.05	1.126	0.016	2.236	0.047
*Position*								
Resident	Ref	Ref	Ref	Ref	Ref	Ref	Ref	Ref
Research	−0.116	−2.053	1.821	0.906	0.351	−1.641	2.343	0.729
Chief Resident	−1.875	−4.021	0.272	0.087	−2.144	−4.322	0.035	0.054
*Native language*								
German	Ref	Ref	Ref	Ref	Ref	Ref	Ref	Ref
French	−1.147	−1.971	−0.324	0.006	−1.269	−2.46	−0.079	0.037
Italian	1.186	0.211	2.161	0.017	0.649	−0.986	2.285	0.436
Spanish	−0.21	−2.399	1.979	0.851	−0.747	−3.054	1.561	0.526
All Others	0.206	−0.656	1.068	0.639	0.04	−1.192	1.271	0.95
Duration of Training	0.013	−0.003	0.029	0.124				
*Training*≥24 M	0.863	0.261	1.465	0.005	0.614	−0.047	1.276	0.069
≤12 M	−0.571	−1.306	0.164	0.128				
12.1–24 M	Ref	Ref	Ref	Ref				
24.1–36 M	−0.018	−1.042	1.006	0.973				
36.1–48 M	−0.559	−1.798	0.68	0.376				
48.1–60 M	−0.376	−2.216	1.464	0.688				
>60 M	−2.19	−3.995	−0.384	0.017				

Adjusted *R*^2^ = 0.031, ANOVA < 0.001

*B-coeff* beta coefficient, *CI* confidence interval, *M* months, *Medschool* country of graduation from medschool, *Ref* reference category

**Table 4:** Uni- and multivariable linear regression analysis of perceived helplessness with sex, age, intended specialty, nationality, country of graduation, position, native language, and duration of training as independent variables

	B-coeff	95% CI		*p*-value	B-coeff	95% CI		*p*-value
		lower	upper			lower	upper	
Male sex	−1.135	−1.613	−0.658	<0.001	−1.154	−1.67	−0.639	<0.001
*Age*	−0.063	−0.133	0.008	0.082				
≤25 years	−0.861	−3.31	1.588	0.491	−0.659	−3.092	1.953	0.658
25.1–30 years	Ref	Ref	Ref	Ref	Ref	Ref	Ref	Ref
30.1–35 years	0.272	−0.698	1.243	0.582	−0.002	−1.012	1.007	0.996
35.1– > 40 years	−0.861	−1.768	0.047	0.063	−0.874	−1.286	0.933	0.755
*Aim*								
Surgery	Ref	Ref	Ref	Ref	Ref	Ref	Ref	Ref
Orthopedics	0.711	−1.298	−0.123	0.018	−0.598	−1.23	0.035	0.064
Urology	−0.326	−1.244	0.592	0.486	−0.05	−1.018	0.919	0.92
Plastic Surgery	−0.256	−1.213	0.702	0.6	−0.279	−1.278	0.72	0.584
Visceral Surgery	−1.241	−2.238	−0.244	0.015	−1.081	−2.094	−0.068	0.036
Hand Surgery	0.99	−0.493	2.472	0.191	0.489	−1.069	2.048	0.538
Pediatric Surgery	−0.49	−1.927	0.947	0.504	−0.545	−1.993	0.902	0.46
Other	−0.247	−1.236	0.743	0.625	−0.056	−1.114	1.003	0.918
*Nationality*								
Swiss	Ref	Ref	Ref	Ref	Ref	Ref	Ref	Ref
German	0.696	0.031	1.36	0.04	0.251	−0.598	1.099	0.562
Austrian	−0.445	−1.559	0.668	0.433	−0.785	−2.018	0.449	0.212
Italian	0.368	−0.492	1.229	0.401	−0.016	−1.504	1.473	0.984
French	−1.502	−2.635	−0.369	0.009	−0.879	−2.261	0.504	0.213
Other/unknown	−0.315	−0.934	0.304	0.319	−0.787	−1.967	0.392	0.191
*Medical school*								
Switzerland	Ref	Ref	Ref	Ref	Ref	Ref	Ref	Ref
Germany	−0.205	−1.097	0.688	0.653	−0.457	−1.427	0.513	0.355
Austria	−0.198	−1.132	0.735	0.677	−0.258	−1.253	0.736	0.61
Italy	−0.11	−1.307	1.088	0.858	0.727	−0.627	2.081	0.292
France	−1.364	−2.574	−0.153	0.027	−0.493	−1.912	0.925	0.495
Others/unknown	0.156	−0.475	0.786	0.629	0.481	−0.435	1.397	0.303
*Position*								
Resident	Ref	Ref	Ref	Ref	Ref	Ref	Ref	Ref
Research	−0.682	−2.2	0.836	0.379	−0.626	−2.21	0.958	0.438
Chief Resident	−1.515	−3.197	0.167	0.077	−1.741	−3.585	0.103	0.064
*Native language*								
German	Ref	Ref	Ref	Ref	Ref	Ref	Ref	Ref
French	−1.483	−2.198	−0.883	<0.001	−1.223	−2.204	−0.242	0.015
Italian	0.003	−0.83	0.721	0.89	−0.183	−1.565	1.199	0.795
Spanish	−1.537	−3.348	0.159	0.075	−1.645	−3.623	0.333	0.103
All Others	−0.269	−1.017	0.363	0.353	0.106	−1.073	1.286	0.859
Duration of Training	0.003	−0.01	0.015	0.674				
*Training *≥24 M	0.375	−0.096	0.847	0.118	0.502	−0.065	1.068	0.082
≤ 12 M	−0.268	−0.846	0.311	0.365				
12.1–24 M	Ref	Ref	Ref	Ref				
24.1–36 M	−0.251	−1.058	0.556	0.542				
36.1–48 M	−0.542	−1.52	0.436	0.277				
48.1–60 M	−0.74	−2.193	0.713	0.318				
>60 M	−2.1	−3.513	−0.687	0.004				

Adjusted *R*^2^ = 0.038, ANOVA *p* < 0.001

*B-coeff* beta coefficient, *CI* confidence interval, *M* months, *Medschool* country of graduation from medschool, *Ref* reference category

**Table 5:** Uni- and multivariable linear regression analysis of perceived self-efficacy with sex, age, intended specialty, nationality, country of graduation, position, native language, and duration of training as independent variables

	B-coeff	95% CI		*p*-value	B-coeff	95% CI		*p*-value
		lower	upper			lower	upper	
Male sex	−0.13	−0.394	0.133	0.331				
*Age*	0.043	0.004	0.083	0.031	0.02	−0.021	0.062	0.336
≤25 years	0.551	−0.819	1.92	0.43				
25.1–30 years	Ref	Ref	Ref	Ref				
30– − 35 years	−0.206	−0.749	0.337	0.456				
35.1–>40 years	0.367	−0.141	0.874	0.157				
*Aim*								
Surgery	Ref	Ref	Ref	Ref	Ref	Ref	Ref	Ref
Orthopedics	−0.417	−0.738	−0.096	0.011	−0.492	−0.829	−0.155	0.004
Urology	−0.867	−1.367	−0.368	<0.001	−0.655	−1.178	−0.132	0.014
Plastic Surgery	−0.291	−0.815	0.234	0.277	−0.534	−1.078	0.01	0.054
Visceral Surgery	−0.815	−1.364	−0.267	0.004	−0.764	−1.32	−0.208	0.007
Hand Surgery	−0.139	−0.939	0.662	0.734	−0.126	−0.965	0.713	0.768
Pediatric Surgery	−0.661	−1.445	0.123	0.098	−0.514	−1.303	0.275	0.201
Other	−0.194	−0.736	0.348	0.483	−0.357	−0.934	0.219	0.224
*Nationality*								
Swiss	Ref	Ref	Ref	Ref	Ref	Ref	Ref	Ref
German	0.002	−0.36	0.365	0.991	−0.212	−0.672	0.247	0.365
Austrian	−0.219	−0.829	0.392	0.483	−0.345	−1.018	0.328	0.315
Italian	0.995	0.526	1.465	<0.001	−0.168	−0.983	0.646	0.685
French	0.358	−0.26	0.976	0.256	0.053	−0.701	0.807	0.89
Other/unknown	0.481	0.143	0.819	0.005	−0.195	−0.836	0.446	0.551
*Medical school*								
Switzerland	Ref	Ref	Ref	Ref	Ref	Ref	Ref	Ref
Germany	−0.0002	−0.486	0.486	1	−0.037	−0.567	0.492	0.89
Austria	−0.115	−0.622	0.391	0.655	−0.259	−0.802	0.284	0.349
Italy	0.835	0.185	1.485	0.012	0.479	−0.258	1.216	0.202
France	0.753	0.089	1.418	0.026	0.567	−0.215	1.35	0.155
Others/unknown	0.664	0.322	1.006	<0.001	0.388	−0.11	0.886	0.127
*Position*								
Resident	Ref	Ref	Ref	Ref				
Research	0.507	−0.332	1.347	0.236				
Chief Resident	−0.368	−1.31	0.574	0.444				
*Native language*								
German	Ref	Ref	Ref	Ref	Ref	Ref	Ref	Ref
French	0.306	−0.053	0.665	0.095	−0.013	−0.55	0.524	0.963
Italian	1.145	0.722	1.568	<0.001	0.922	0.164	1.681	0.017
Spanish	1.308	0.353	2.263	0.007	0.665	−0.413	1.744	0.226
All Others	0.457	0.081	0.833	0.017	0.242	−0.401	0.884	0.461
Duration of Training	0.011	0.003	0.018	0.003				
*Training *≤24 M	0.134	0.251	0.777	<0.001	0.292	−0.013	0.598	0.06
≤12 M	−0.327	−0.643	−0.01	0.043				
12.1–24 M	Ref	Ref	Ref	Ref				
24.1–36 M	0.245	−0.196	0.686	0.275				
36.1–48 M	−0.032	−0.567	0.502	0.905				
48.1–60 M	0.348	−0.446	1.142	0.39				
>60 M	0.002	−0.777	0.781	0.996				

Adjusted *R*^2^ = 0.031, ANOVA *p* < 0.001

*B-coeff* beta coefficient, *CI* confidence interval, *M* months, *Medschool* country of graduation from medschool, *Ref* reference category

## Discussion

This study reported the first nationwide analysis on perceived stress levels, helplessness, and self-efficacy in surgical residencies, using an established and validated instrument and a representative sample of Swiss surgical residents queried over 5 years [42–44]. Two North American studies [45, 46] investigated stress levels in vascular and general surgeons, yet these reports are not representative of US surgical residencies at large. To the best of our knowledge, there are no similar studies available for European surgical residents.

The demographics of the present cohort match the overall medical demographics in Switzerland. Indeed, in 2020 practicing physicians in Switzerland (all medical specialties) were 56% male and 44% female, whereas 37.4% of the Swiss medical workforce graduated from a foreign medical school. Gender balance is roughly similar to the figures reported by most countries of the Organisation for Economic Cooperation and Development, but for the share of international medical graduates (IMG) for which Switzerland tops all ranking with 37.4% IMG, compared to 26% IMG in the USA, 11.5% in France and Germany, and 9.4% in Denmark [47, 48].

Chronic stress has a proven negative effect on health, performance, and learning [36, 49, 50]. Suboptimal working and learning environment have a negative impact on workplace efficiency, resident’s mental condition, and attrition [22, 51–54]. The present study reported markedly higher level of negative stress and helplessness in surgery residents when compared to the general population. The even worse scores demonstrated by female residents are a further major concern, as women represent the majority of medical graduates in many countries and a growing share of surgical residents. Indeed, 43.5% of the Swiss surgical residents in the present study were women. A study investigating first- and second-year residents workplace experience in the early 2000 in Switzerland already reported higher levels of effort–reward imbalance in females and in surgical specialties, than in males and in medical specialties [55]. In an American longitudinal study of surgical residents, female sex was the independent factor most strongly associated with residency attrition [56], while a meta-analysis including 19,821 American residents revealed an overall attrition prevalence of 18%, affecting predominantly female residents (25% vs. 15% in males, *p* = 008), whereas half of the residents left after their first postgraduate year [57]. In prior reports across different industries and countries, women also reported higher stress scores than males [16, 35, 58], in agreement with the present study. Hence, the present results are alarming at a time where many countries struggle to maintain their surgical workforce, in the Western world and in the developing world alike.

Switzerland is an immigration country with every fifth person living in the country not holding Swiss citizenship [36] and a high dependence of Swiss health care toward foreign healthcare workers, as illustrated by the fact that 37.4% of the Swiss medical workforce did not graduate in Switzerland [48]. In surgical residencies, dependence toward IMG is even greater: While in the prior decade 2002–2012 [59], a slight majority of surgical residents (55%) graduated from a Swiss medical school, in the present cohort the share of Swiss graduates decreased to 38.7% and seems to stabilize around 40% if one takes into account the data from the 2021 Surgical Basic Exam. In this context, the observed vulnerability of Italian nationals to stress, who represent 10% of the present cohort, is another concern, shared by foreign medical graduates not coming from a country adjacent to Switzerland. Indeed, migrant workers are confronted to acculturative stress in most industries and countries [60].

These findings should be addressed to improve medical training environments and create more supportive and inclusive residency programs. In this context, stress coping and stress reduction initiatives may prevent burnout and resident attrition, whereas these initiatives should be preferentially directed toward female and foreign residents [61, 62].

Earlier studies have classified stressors at medical school and residency into three main categories: academic pressures, social factors including age, sex, ethnicity, and relationship status, and financial problems [33, 36, 37, 40, 49, 62, 63]. This study found no association with age, while country of graduation and foreign mother language negatively affected stress level. Relationship and parental status were not investigated in the present study. US studies of residents’ well-being have shown that parenthood affected stress levels of surgical residents, women more than men, and this important point deserves further investigation and attention [61, 64–66]. Providing day care opportunities compliant with working schedules of residents, and shared parental responsibilities that allow a father to grow in his role are obvious steps, dearly requested yet rarely witnessed [61, 66].

Financial data were not collected, although debt and financial stress have been associated with attrition from surgical residency [67]. Yet, debt is uncommon for Swiss medical graduates as Swiss medical schools are virtually tuition-free while student grants are largely available to cover cost of living when needed. Furthermore, the average entry salary of a resident is higher than the median income in Switzerland, allowing for a stress-free living, in financial terms at least. Higher income and purchasing power in Switzerland is frequently reported as one of the reasons for an international medical graduate to seek employment and residency in Switzerland [68, 69].

Proper information of incoming residents on the structure, requirements, and overall organization of the residency programs is one of the easier actions to undertake with the potential to better align expectations with reality and avoid deception and attrition [70]. Mentorship and physician assistance programs are a further way to improve residency: They can be used to attract medical student toward surgical specialties and most importantly to retain residents in training. Structured assistance and counseling aimed at improving work–life balance, supporting family life, increasing access to mental health support, and supporting career transitions have improved residents’ attrition rate and well-being [71, 72].

While the present study was nationwide and hence representative, it did not investigate in more details further potential stressors in residency that may account for the higher level of stress reported. As discussed above, such a detailed analysis was performed in medical schools students [18, 45] and some of the reported student stressors could be investigated in the present study. Also, possible differences between academic and non-academic residency programs were not investigated, whereas prior reports suggested a higher attrition rate in academic institutions and during a research year [55, 73–75].

While internal consistency of the PSS-10 instrument was demonstrated, response and time bias may have affected self-reported measures. Yet, the magnitude of the differences presently measured and the focus of the PSS instrument over a 4-week period more than on a given point of time should minimize this possible bias.

Last, some of the respondents did repeat the Surgical Basis Exam and this could possibly affect the perceived stress levels. While the vast majority of the respondents were first takers within 2 years of residency training, full anonymization prevents a separate analysis of examination retakers. Yet, the influence of time spent in training was accounted for in uni- and multivariable analysis, with no statistical difference by postgraduation year when compared to the reference population of residents within the first 2 years of residency.

Further studies should investigate with greater granularity the working and learning environments of surgical residents in order to better understand and improve perceived stress levels. Female residents deserve particular attention in this matter, owing to the importance of the female population and to the stronger impact of the environment on the stress levels reported by women.

## Conclusion

Perceived stress levels are high in both sexes in this large, prospective, and representative cohort study of Swiss surgical residents in their early years of training. This should warrant intervention, as chronic stress is detrimental and associated with burnout and residency attrition, in particular in the first 2 years of training [57, 73, 76, 77]. These worrisome figures may directly contribute to the declining attractivity of surgical residencies, as it was suggested in a previous survey of residents’ reasons for specialty choice [78, 79]. Importantly, sex differences were remarkable and should be further addressed. Females endured significantly worse stress and helplessness levels than males. IMG, representing a large group of surgical residents, were also struggling to adapt to surgical residencies and deserve dedicated attention.

Detailed analysis and correction of stressors are urgently needed to improve residency programs.
